# Some Non-Combatant Responsibilities

**Published:** 1914-08-22

**Authors:** 


### SOME NON-COMBATANT RESPONSIBILITIES.

One of the most encouraging features of the present situation is the recognition, practically universal throughout the country, of the claim of national duty. There is for all of us something to do apart from the cultivation of our individual interests. If the demand seems most conspicuous in the case of those who are summoned to active service, it is not less real for those who remain at home. In both of these groups the medical profession has its representatives. The members of the Navy and Army medical services take their positions with their fellow sailors and soldiers, and all our goodwill and confidence are with them. Private practitioners stand on a somewhat different footing, but none the less are they animated by public spirit and by a resolve, without display and without ostentation, to serve the national cause.

First among these stand members of the Territorial forces who, on a brief summons, have yielded their civil duties on the call to mobilise. In many instances this has meant the sacrifice of work which is the livelihood of themselves and of their wives and families. Often it has been quite impossible in such circumstances to make adequate and satisfactory provision for the conduct of their practices during their absence. This absence may be a prolonged one, and from it they run the danger of considerable loss and even of ruin. It is for their brethren to see that such consequences do not follow. No practitioner who has left his work at the call of his King and country must be allowed to carry a burden of anxiety in relation to his means of making a living. He must be sustained by the conviction that when in due time he returns to civil life he will find his practice conserved and ready to receive him. In many districts already steps have been taken to secure this. Those who remain are agreeing to act as friendly substitutes for those who have gone. It is a good and loyal undertaking, in harmony with the best professional traditions and with the sanctity of patriotic obligation. We are convinced that it will be generally observed.

There is one aspect of this question, which is perhaps a somewhat delicate one, but on which we will venture to say a word. It is useless to attempt to hide the fact that the National Insurance Act has divided the profession into two camps, and that a feeling not free from bitterness has been excited between the disputants. We trust it is possible to banish this, at least for the time being. The politicians have set us a splendid example, and this is no occasion for domestic quarrels. No one asks that strong convictions and opinions shall be permanently abandoned. If necessary, they can be resumed when national peace reasserts its sway. But in the meantime we plead for mutual help independently of whether we have opposed or favoured the panel. Let no man object because of a difference of opinion to serve the interests of a colleague called from his civil work by the claims of military obligation.

Another opportunity which offers itself to the medical practitioner is presented by the position of women and children whose husbands and fathers have been called out with the Reserves. Many of these are left with but scanty provision. Of their special and abiding anxieties we say nothing. We cannot doubt that such a position will appeal to members of the medical profession, and that it will be met by ready and willing consideration and treatment apart from all question of professional fees. Sooner or later, possibly, there may be other families where the income has failed in consequence of loss of employment as a result of the war. The appeal here is not less strong, and we commend it with confidence to all our readers.

A word may be allowed on the exercise of the practitioner's personal influence in the direction and maintenance of public opinion. His position and his associations give him great opportunities, and he may readily prove himself a sustaining force of no small value. Undoubtedly the nation generally has shown itself calm and resolved. It is necessary that this attitude be continued. There are two influences which oppose it—the circulation of unfounded reports and rumours, and the possibility of reverses to our arms. Against the repetition of mere club gossip and chatter every educated man should set his face as a flint, and if misfortunes, more or less severe, come to us, there is the greater demand for the equal mind. Every individual who, by example especially, helps to suppress exaggeration, to discountenance panic, and to keep high the national resolve, is, in so doing, serving well his country's cause. In all these directions the medical practitioner has the opportunity for much quiet but effective patriotic service.

